# Single cell transcriptomic analysis of prostate cancer cells

**DOI:** 10.1186/1471-2199-14-6

**Published:** 2013-02-16

**Authors:** Christopher J Welty, Ilsa Coleman, Roger Coleman, Bryce Lakely, Jing Xia, Shu Chen, Roman Gulati, Sandy R Larson, Paul H Lange, Bruce Montgomery, Peter S Nelson, Robert L Vessella, Colm Morrissey

**Affiliations:** 1 Department of Urology, University of Washington, Seattle, WA, USA; 2 Fred Hutchinson Cancer Research Center, Seattle, WA, USA; 3 Department of Veterans Affairs Medical Center, Seattle, WA, USA; 4 Department of Medicine, University of Washington, Seattle, WA, USA; 5 Genitourinary Cancer Research Laboratory, Department of Urology, University of Washington, Box 356510, Seattle, WA, 98195, USA

**Keywords:** Prostate cancer, Single-cell, Transcriptome, Disseminated tumor cells

## Abstract

**Background:**

The ability to interrogate circulating tumor cells (CTC) and disseminated tumor cells (DTC) is restricted by the small number detected and isolated (typically <10). To determine if a commercially available technology could provide a transcriptomic profile of a single prostate cancer (PCa) cell, we clonally selected and cultured a single passage of cell cycle synchronized C4-2B PCa cells. Ten sets of single, 5-, or 10-cells were isolated using a micromanipulator under direct visualization with an inverted microscope. Additionally, two groups of 10 individual DTC, each isolated from bone marrow of 2 patients with metastatic PCa were obtained. RNA was amplified using the WT-Ovation™ One-Direct Amplification System. The amplified material was hybridized on a 44K Whole Human Gene Expression Microarray. A high stringency threshold, a mean Alexa Fluor® 3 signal intensity above 300, was used for gene detection. Relative expression levels were validated for select genes using real-time PCR (RT-qPCR).

**Results:**

Using this approach, 22,410, 20,423, and 17,009 probes were positive on the arrays from 10-cell pools, 5-cell pools, and single-cells, respectively. The sensitivity and specificity of gene detection on the single-cell analyses were 0.739 and 0.972 respectively when compared to 10-cell pools, and 0.814 and 0.979 respectively when compared to 5-cell pools, demonstrating a low false positive rate. Among 10,000 randomly selected pairs of genes, the Pearson correlation coefficient was 0.875 between the single-cell and 5-cell pools and 0.783 between the single-cell and 10-cell pools. As expected, abundant transcripts in the 5- and 10-cell samples were detected by RT-qPCR in the single-cell isolates, while lower abundance messages were not. Using the same stringency, 16,039 probes were positive on the patient single-cell arrays. Cluster analysis showed that all 10 DTC grouped together within each patient.

**Conclusions:**

A transcriptomic profile can be reliably obtained from a single cell using commercially available technology. As expected, fewer amplified genes are detected from a single-cell sample than from pooled-cell samples, however this method can be used to reliably obtain a transcriptomic profile from DTC isolated from the bone marrow of patients with PCa.

## Background

The development of novel technologies for capturing, enriching, and preserving exfoliated abnormal cells from body fluids or effusions as well as methods for concentrating the tumor-derived sub-cellular material for use in biomarker studies is currently a focus in the cancer field. Our laboratory and others have previously noted the presence of circulating tumor cells (CTC) in the blood and disseminated tumor cells (DTC) in the bone marrow of prostate cancer (PCa) patients at time of radical prostatectomy, in post-radical prostatectomy patients with no evidence of disease, and in patients with advanced disease [1-4]. While men with DTC in the bone marrow are at an increased risk of future disease recurrence, many will not recur [2]. In addition, PCa is somewhat unique in the long delay that is often seen between the treatment of localized disease and the development of metastases. This suggests that tumor cells can remain dormant in the bone marrow for years and are subsequently “activated” by unknown mechanisms in some patients, leading to the formation of metastases [5-7]. However, to date there are no known markers to separate recurrent from indolent CTC/DTC or to distinguish dormant from senescent cells.

Transcriptomic analysis of CTC/DTC possesses the potential to identify markers that distinguish recurrent from indolent disease and genes important for tumor dormancy. However, using standard techniques this approach is limited by the need to pool multiple DTC to obtain enough mRNA for amplification [1,2,5]. This approach may combine dormant and indolent DTC, making identification of dormancy markers difficult or impossible. The primary challenge of single-cell transcriptome analysis is amplifying the mRNA to a detectable level while maintaining the correct sequences and relative signal intensities of the expressed genes.

Several methods for single-cell transcriptome analysis have been described. However, these methods are limited in their potential application due to the requirement for high throughput, time constraints, monetary considerations, and reproducibility. Therefore, we aimed to develop and validate an efficient, reproducible method for obtaining a transcriptomic profile from a single cell using commercially available technologies.

All single-cell transcriptome profiling methods rely on the creation and amplification of a cDNA library. To be detected using current technology, available mRNA from a single cell must be amplified approximately 10^7^-fold. There are two amplification strategies used: linear amplification through *in vitro* transcription (IVT) and exponential amplification through a PCR-based method [8-11]. The IVT method is more stringent and reduces the number of non-specific byproducts formed during amplification, but it is time intensive as each round of IVT only amplifies the available cDNA approximately 1000-fold. Through exponential amplification, PCR-based methods are more time efficient but are challenging to use with low abundance mRNA due to the potential for amplification of primer-primer dimers and loss of relative signal intensity through multiple rounds of amplification.

In the method described here, a commercial technology is used for amplification of low-abundance mRNA and a commercially available human oligonucleotide microarray to profile the transcriptome from a single PCa cell. Using clonally selected, synchronized single C4-2B PCa cells, pools of 5 cells, and pools of 10 cells, we determined that this method is efficient and effective but is limited predominantly by the abundance of the mRNA species available for amplification from a single cell. Herein we describe the usefulness and limitations of this approach.

## Methods

### Culture and isolation of individual PCa cells

To obtain a synchronized PCa cell population for analysis, we clonally selected C4-2B cells and cultured a single passage of cells in RPMI 1640 medium (Life Sciences Technologies Inc.) with 10% FBS. Cells were treated with 30 mg/ml of Aphidicolin (Sigma) 24 h prior to isolation. Cells were trypsinized and resuspended in RPMI 1640 with 10% FBS. Ten replicates of single, pools of 5, and pools of 10 cells (a total of 30 samples) were isolated with glass micropipettes using a TransferMan® NK micromanipulator (Eppendorf), lysed in a 2 μl drop of WT-Ovation™ One-Direct Amplification System lysis buffer (NuGEN) on a siliconized glass slide, and then stored for a minimum of 2 weeks at −80°C before amplification. The transfer of cells to lysis buffer was verified by direct visualization.

### Isolation of individual DTC from the bone marrow of PCa patients

All materials were acquired and used conforming with IRB-approved protocols at the University of Washington. DTC were isolated from bone marrow samples of PCa patients with advanced disease. Ten ml of bone marrow was aspirated from the iliac crest into a 30 ml syringe containing 10 ml of 6% sodium citrate. In samples obtained from patients, bilateral aspirates were obtained and combined for a total of 20 ml of bone marrow. Samples were obtained using local anesthesia and taken from the posterior iliac crest. Processing of samples commenced within 1–2 hours and was completed within 5 hours.

### Cell enrichment

Cell enrichment and isolation was performed as previously described [12]. Briefly, bone marrow aspirates were placed over a 15 ml volume of Ficoll-Isopaque 1.077 g/ml (Accurate Chemical, Westbury, NY). Centrifugation subsequently yielded a mononuclear cell layer containing DTC, if present. The MACS system for immunomagnetic selection (Miltenyi Biotec, Auburn, CA) was then employed. Anti-CD45 and anti-CD61 antibodies were used for negative selection, targeting leukocytes, megakaryocytes, and platelets. Positive selection was then performed with immunomagnetic beads coated with anti-human epithelial antigen (HEA) antibodies.

### Identification of DTC

For identification and isolation of DTC, the enriched population was subjected to immunostaining with fluorescein isothiocyanate labeled anti-BerEP4 antibodies (Dako, Carpinteria, CA) which bind a different epitope on HEA than the anti-HEA antibody used for positive selection. A phycoerythrin conjugated anti-CD45 antibody was also added for identification of leukocytes. The cells were kept on ice and viewed under fluorescent light using an inverted microscope. Individual cells were isolated using a micromanipulator, placed in 2 μl of lysis buffer from the WT-Ovation™ One-Direct Amplification System (NuGEN) and stored at −80°C.

### Amplification of total RNA from PCa cells

Total RNA was amplified from each sample using the WT-Ovation™ One-Direct Amplification System (NuGEN) according to the manufacturer’s directions. The use of an aluminum cooling block on ice facilitated the handling of the reaction tubes. The amplified cDNA product purity and yield was determined by measuring the product’s absorbance at 260, 280, and 340 nm. The size distribution of the amplified cDNA product was analyzed on an Agilent Bioanalyzer using the RNA 6000 Pico LabChip with the mRNA Pico program. The mean fragment size was determined by creating a fragment on the electropherogram beginning at the start of the fluorescent signal and ending at a point corresponding to 50% of the total area. The end size (nt) value for this point was used to represent the mean fragment size. Post-SPIA modification and post-amplification work was performed in a separate workspace.

### Real-Time PCR (RT-qPCR)

Amplified cDNA from each sample was used for RT-qPCR amplification of 10 genes (Additional file 1: Tables S1). We measured relative gene expression changes in triplicate reactions using 5 ng cDNA, 0.2 μmol/L of each primer, and Power SYBR Green PCR master mix (Applied Biosystems) in a reaction volume of 10 μL on a 7900HT Real-Time PCR machine (Applied Biosystems). All reactions were run in triplicate and assessed for quality and specificity by analysis of dissociation curves. We normalized mean quantification cycle value (Cq) for each gene to a housekeeping gene, RPL13A. We identified poor quality samples using Dixon’s Q test, a non-parametric test appropriate for small samples, to identify outliers based on the means of triplicate gene expression changes of four housekeeping genes (ACTB, RPL13A, YWHAZ, and GAPDH) [13].

### Labeling and hybridization of amplified material on Agilent chip

Amplified cDNA from each sample and reference pool were labeled using the BioPrime® Total Genomic Labeling System (Invitrogen™). The reference pool was prepared by combining equal quantities of total RNA isolated from LNCaP, DU145, PC3, and CWR22 prostate epithelial cell lines grown at log phase, amplifying through two rounds using the MessageAmp™ II aRNA Amplification Kit (Ambion®), and converting to first strand cDNA. Hybridization probes were prepared by combining 6 mg of Alexa Fluor® 3 labeled sample and 1 mg Alexa Fluor® 5 labeled reference and denatured at 95°C and hybridized at 63°C on Agilent Human 4x44K microarrays and processed following the manufacturer’s specifications. Arrays were scanned on an Agilent DNA Microarray Scanner.

### Gene expression analysis

Graphical examination of raw array signal indicated a batch effect due to subtle day-to-day variability in sample processing. We corrected for this effect by separately estimating mean Alexa Fluor® 3 and mean Alexa Fluor® 5 signal within single-, 5-, and 10-cell arrays between experimental days and adjusting values on the second day by the estimated difference. We normalized day-effect-corrected signals within arrays using loess and normal-exponential background correction with offset 50 and quantile normalized between arrays separately for single-, 5-, and 10-cell arrays using the Limma package in R [14]. We then calculated sensitivity and specificity for the single-cell arrays using both the 5- and 10-cell arrays as the gold standard. Sensitivity is the proportion of commonly expressed probes in both array types out of total number of expressed probes in the gold standard. Specificity is the proportion of commonly unexpressed probes in both array types out of total number of unexpressed probes in the gold standard. Confidence intervals are based on a Bayesian approach using a uniform prior distribution. The same analysis was done for arrays from 5-cell pools using arrays from 10-cell pools as the gold standard. Relative signal intensity of individual markers was assessed using the M-ratio, defined as log_2_ (Alexa Fluor® 3/Alexa Fluor® 5), for all probes detected on all three sets of arrays, with detection defined as across-array-averaged, day-effect-adjusted, normalized Alexa Fluor® 3 signal greater than 300. We calculated Pearson correlation of the M-ratios between the single- and 5-cell, single- and 10-cell, and 5- and 10-cell arrays. To assess the ability of the single-cell arrays to accurately order gene expression levels, we also calculated Spearman correlation of the ranks of M-ratios. The ability of the single-cell arrays to accurately detect the relative expression of probes on the same array was assessed by examining Pearson correlation of ratios of M-ratios from 10,000 randomly selected probes between each pair of arrays. Gene set enrichment analysis was applied for a select set of genes in the single-, 5-, and 10-cell arrays using the mean centered log_2_ signal intensities [15,16].

## Results

### Quality control

**Figure 1:**
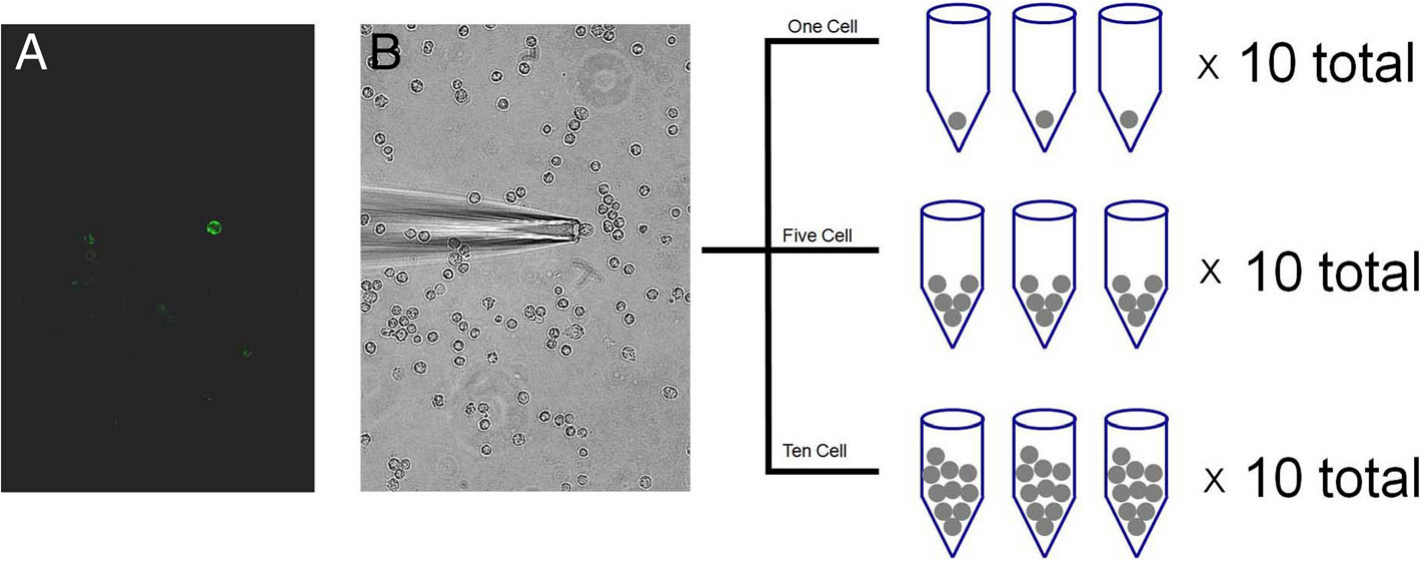
**Isolation of individual cells.A**. Fluorescently labeled EpCAM positive cells are identified and **B**. Under light microscopy a single C4-2B cell is harvested using a micropipette system (40X).

Cultured C4-2B cells, a PCa line derived from the LNCaP cell line, were grown, isolated, and amplified as illustrated (Figure 1). Quality of the amplified genetic material was assessed in four ways: purity and yield by spectrophotometer absorbance, gene detection by RT-qPCR, efficiency of labeling amplified material prior to microarray hybridization, and standard deviation of oligo hybridization to microarrays. The range of Abs_260_/Abs_280_ ratios of all samples was found to be 1.9 to 2.0, indicating high purity. The average amplified cDNA product yield for single-cell samples was 12.1 +/− 1.9 μg, for 5-cell samples 12.6 +/− 1.8 μg, and for 10-cell samples 13.5 +/− 2.3 μg. Yields are not expected to be directly proportional to input amount because, in the absence of template, one would expect nonspecific cDNA yields below 4 to 5 μg of product. Using a Dixon’s Q test we determined that there were no outliers for the single-, 5-, and 10-cell sample yields. Additionally, mean fragment sizes of the amplified products assessed on a Bioanalyzer were not significantly different between 1-, 5- and 10-cell samples. RT-qPCR was performed for 10 genes for all amplified samples. Specific genes and primers used are listed in Additional file 1: Table S1. Four housekeeping genes (ACTB, RPL13A, YWHAZ, and GAPDH), were reliably detected in the single-, 5-, and 10-cell samples. Based on the RT-qPCR results, we determined that one of the single-cell samples and one of the 10-cell samples was a failure (Additional file 2: Table S2). In addition, we determined that the Cq for individual cells for housekeeping genes was higher than that of 10 pg of RNA from the same clonal population suggesting less material was available for amplification from a live single cell isolated from culture (Additional file 2: Table S2). For microarray analysis, amplified cDNA from the single-, 5-, and 10-cell samples and from a reference pool were labeled using the BioPrime® Total Genomic Labeling System (Invitrogen™). Labeling efficiency was defined as the amount of Alexa Fluor® 3 label used of amplified RNA. Labeling efficiency was 5.2 ± 1.4 pmol/ng for the single-cell samples, 6.3 ± 1.7 pmol/ng for the 5-cell samples, and 8.6 ± 1.6 pmol/ng for the 10-cell samples. This indicates that RNA quality was similar for all three samples, but labeling efficiency was higher with larger numbers of cells. Once again, a Dixon’s Q test was used to identify potential outliers based on labeling efficiency and no significant outliers were found. To determine if poor quality samples could be identified based on Alexa Fluor® 3 intensity, we analyzed the changes of distributions of log2(Alexa Fluor® 3) for the 10 replicates in the single-, 5-, and 10-cell samples. Using a Dixon’s Q test based on 25th, 50th, and 75th percentiles, maximum values, and mean absolute deviations to quantify array performance, we did not identify additional poor quality samples. The samples deemed to be of poor quality by any of the four criteria (purity and yield by spectrophotometer absorbance, gene detection by RT-qPCR, efficiency of labeling amplified material prior to microarray hybridization, or the standard deviation of oligo hybridization to microarrays) were excluded from further analyses.

### Microarray results

**Figure 2:**
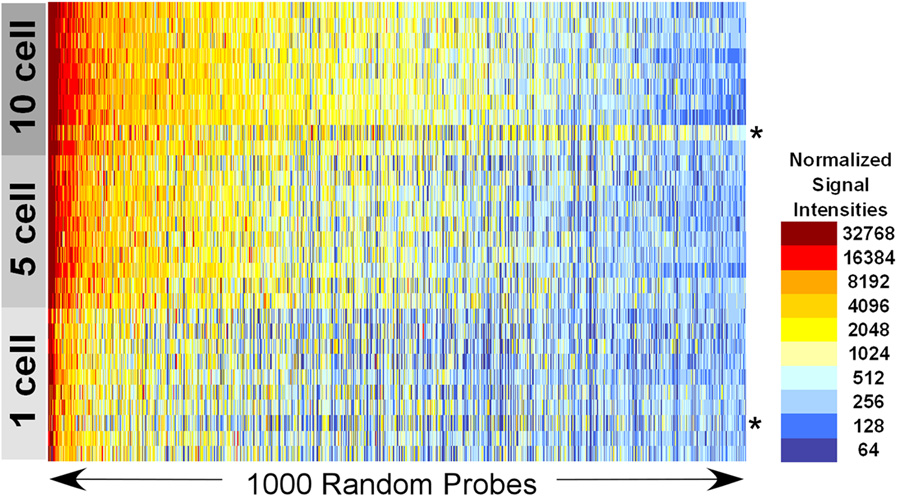
**Microarray results.** Heat map representing magnitude of Alexa Fluor® 3 signal intensity levels. *Indicates removed from analysis based on quality control.

Amplified and Alexa Fluor® 3 labeled single-, 5-, and 10-cell samples were hybridized to Agilent 4x44k human genome microarrays along with Alexa Fluor® 5 labeled reference RNA (Figure 2). A total of 38,695 probes were present on each array. Previously, we obtained 24,885 positive probes on a reference array, which represents the average number of probes detected from C4-2B cells using standard mRNA isolation, amplification, and array protocols. In this study, 17,009 probes were positive on the single-cell arrays, 20,423 probes on the arrays from 5-cell pools, and 22,410 probes on the arrays from 10-cell pools. The sensitivity and specificity of gene detection on the arrays from single-cell pools were 0.739 and 0.972 respectively when compared to arrays from 10-cell pools, and 0.814 and 0.979 respectively when compared to arrays from 5-cell pools, demonstrating a low false positive rate.

### Relative signal intensity

**Figure 3:**
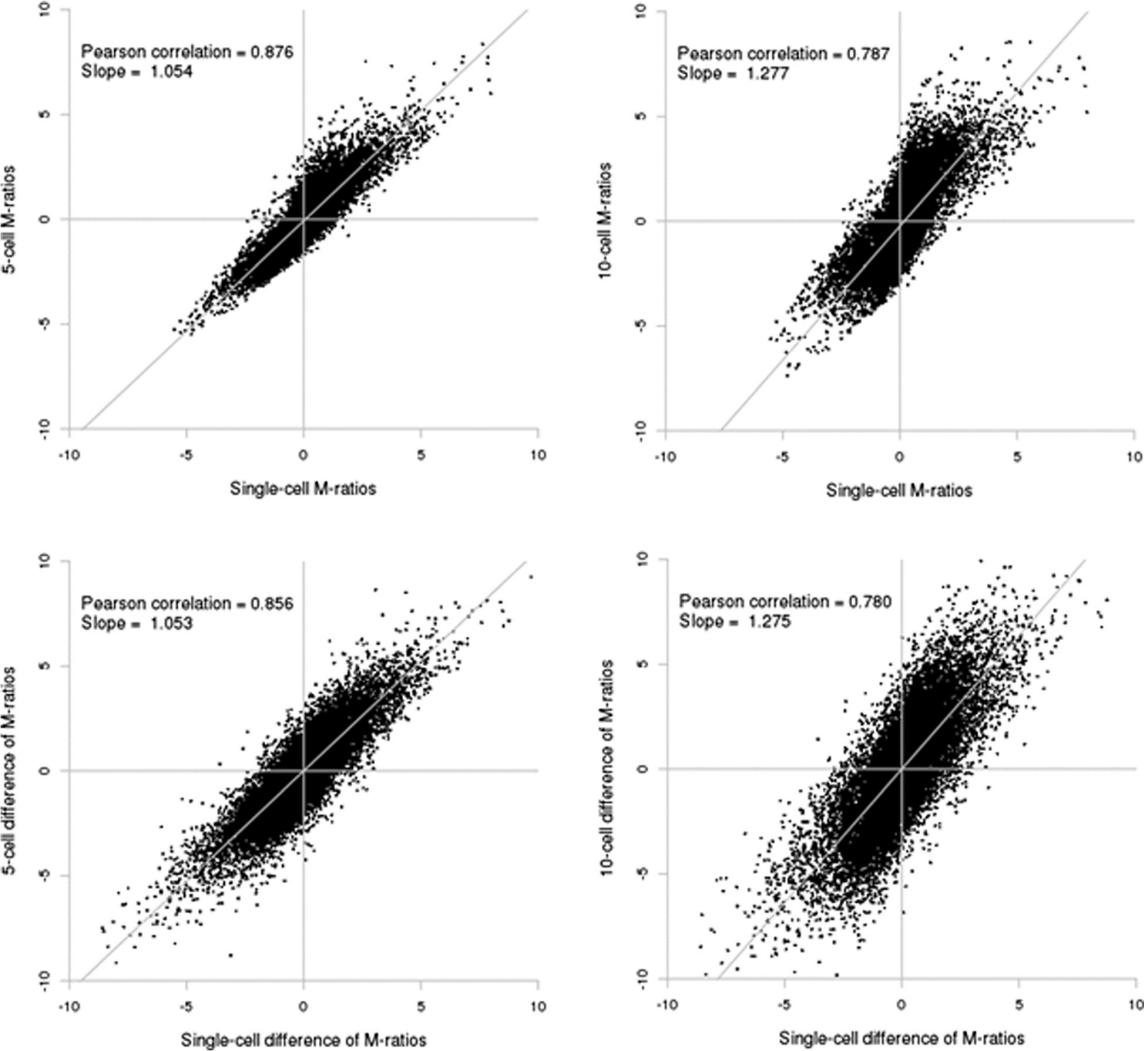
**Correlation of M-ratios (top two panels) and difference of M-ratios (bottom two panels) between arrays.** The mean M-ratio for each probe detected on the single-cell arrays was compared to the M-ratio for the same probe on the 5- or 10-cell array. The M-ratio is defined as log_2_ (Alexa Fluor® 3/Alexa Fluor® 5).

The Pearson correlation between M-ratios on the arrays from single-cell and 5-cell pools was 0.876 and on the arrays from single-cell and 10-cell pools was 0.787. The Spearman correlation between ranks of M-ratios on the arrays from single-cell and 5-cell pools was 0.842 and on the arrays from single-cell and 10-cell pools was 0.786. To test whether gene-to-gene ratios were maintained across sample type we randomly selected 10,000 pairs of genes as an approximation to all 133 million pairs of genes, which we refer to as differences of M-ratios. The Pearson correlation of differences between M-ratios on the arrays from cells single and 5-cell pools was 0.856 and on the single-cell and 10-cell pools was 0.780. Figure 3 shows the global correlation of M-ratios and difference of M-ratios between single-cell and 5-cell and single-cell and 10-cell pools. We also conducted an M-ratio pair-wise correlation analysis for the single cell to gauge the presence of outliers. Based on the correlation analysis, we could not find any outliers for the single cells (Additional file 3: Table S3). The strong positive correlations between M-ratios, ranks of M-ratios, and ratios of M-ratios on the single-cell analyses relative to the 5- and 10-cell pools indicates that signal intensities, their ordering, and their relative levels are generally preserved in the arrays from single-cells. Furthermore the average 10-cell profile versus each individual cell profile the pair-wise correlation coefficient ranged from 0.537 to 0.787, suggesting limited variability between the 1 cell profiles.

### RT-qPCR results

**Figure 4:**
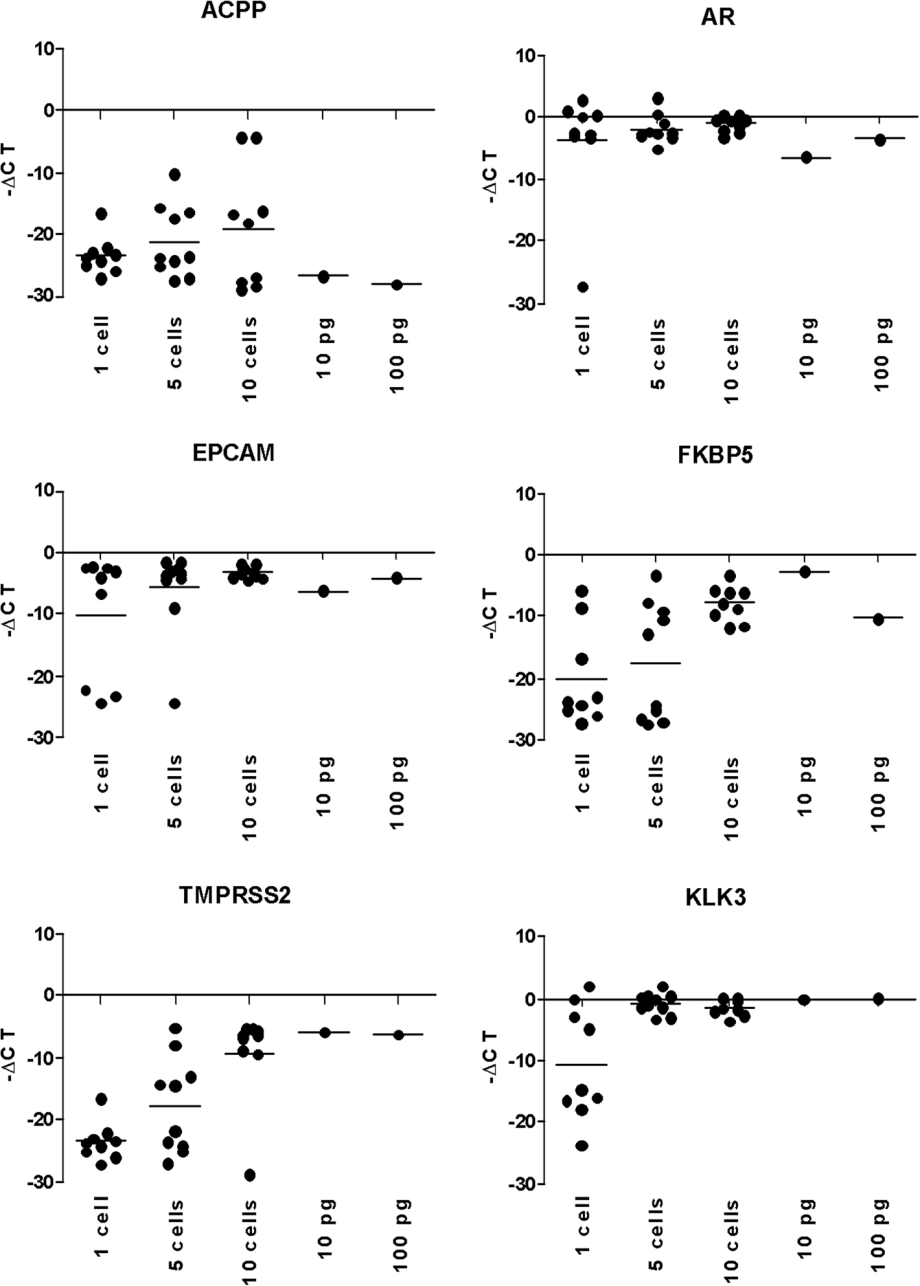
**Real-time PCR results.** RT-qPCR results for AR, EPCAM, KLK3, TMPRSS2, FKBP5 and ACPP. Inverse cycle thresholds relative to RPL13A are shown for single-cell, 5-cell, 10-cell samples as well as 10 pg and 100 pg and unamplified of C4-2B total RNA from the same original culture.

To determine if RT-qPCR could validate the results of the gene expression arrays, we assessed the expression of one epithelial (EPCAM) and five well known prostate epithelial associated genes. We compared the expression levels relative to RPL13A in each of the single-, 5-, and 10-cell samples as well as 10 and 100 pg of C4-2B RNA from the same culture that the other C4-2B cells were isolated from (Figure 4). For the androgen receptor (AR), epithelial cell adhesion molecule (EPCAM), and prostate-specific antigen (KLK3), we observed consistency between the single-, 5-, and 10-cell samples. However, there was a decrease in expression levels for TMPRSS2, FK506-binding protein 5 (FKBP5), and prostatic acid phosphatase (ACPP) respectively in the single-cell samples relative to the 5- and 10-cell samples. Additionally FKBP5 was not detected in five 5-cell samples and ACPP was not detected in six 5-cell samples and four 10-cell samples (Figure 4).

### Comparison of microarray signal intensity to RT-qPCR

We wished to determine an approximate cut off that would indicate when there is a loss of fidelity between the oligo gene expression array Alexa Fluor® 3 signal intensity and RT-qPCR. Our results suggest that an Alexa Fluor® 3 signal intensity above the 75^th^ percentile would be an approximate cut off that we could use to validate our results using RT-qPCR for the C4-2B samples, recognizing that there is inconsistency between the oligo labeling on the array and the amplicons obtained using RT-qPCR (Tables 1 and 2). Nonetheless this cutoff is a useful indicator of fidelity.

**Table 1:** Detection of six selected genes by RT-qPCR from a total of 9 samples in the 1 and 10 cell category and 10 samples in the 5 cell category

	**# of samples detected by RT-qPCR**			**Rank percentile (n = 38695 probes)**		
**Gene**	**1-cell (n = 9)**	**5-cell (n = 10)**	**10-cell (n = 9)**	**1-cell (n = 9)**	**5-cell (n = 10)**	**10-cell (n = 9)**
AR	8	10	9	99	99	99
KLK3	6	10	9	94	98	95
EPCAM	6	9	9	92	95	95
TMPRSS2	0	4	8	85	89	86
FKBP5	2	5	9	69	60	63
ACPP	0	4	5	50	45	54

Detection was considered as having an inverse Cq of at least −20 relative to RPL13A.

**Table 2:** Detection of six selected genes by RT-qPCR from a total of 9 samples in the 1 and 10 cell category and 10 samples in the 5 cell category

	**# of samples detected by RT-qPCR**			**Rank percentile (n = 38695 probes)**		
**Gene**	**1-cell (n = 9)**	**5-cell (n = 10)**	**10-cell (n = 9)**	**1-cell (n = 9)**	**5-cell (n = 10)**	**10-cell (n = 9)**
ACTB	7	7	9	100	100	100
GAPDH	8	10	9	94	94	90
YWHAZ	8	10	9	97	98	98
RPL13A	9	10	9	99	100	100

Detection was considered as having a Cq of <30. Rank percentile represents the average Alexa Fluor® 3 intensity from all oligonucleotides on the array that passed quality control for a given gene with outliers excluded using Dixon’s Q test. n = number of samples analyzed.

### Gene set enrichment analysis

To determine which biological pathways were consistent between the single-, 5-, and 10-cell C4-2B samples, we performed gene set enrichment analysis. The top 25 biological pathways are listed in Additional file 4: Table S4 with a FDR q-value < 0.0001. As expected, the top 25 pathways are associated with RNA processing, protein translation, and mitochondrial activity. The Pearson correlations for the normalized enrichment scores were significant at 0.932 and 0.923 for single- versus 5- and 10-cell samples respectively and 0.946 for 5- versus 10-cell samples.

### The transcriptomic analysis of patient DTC

**Figure 5:**
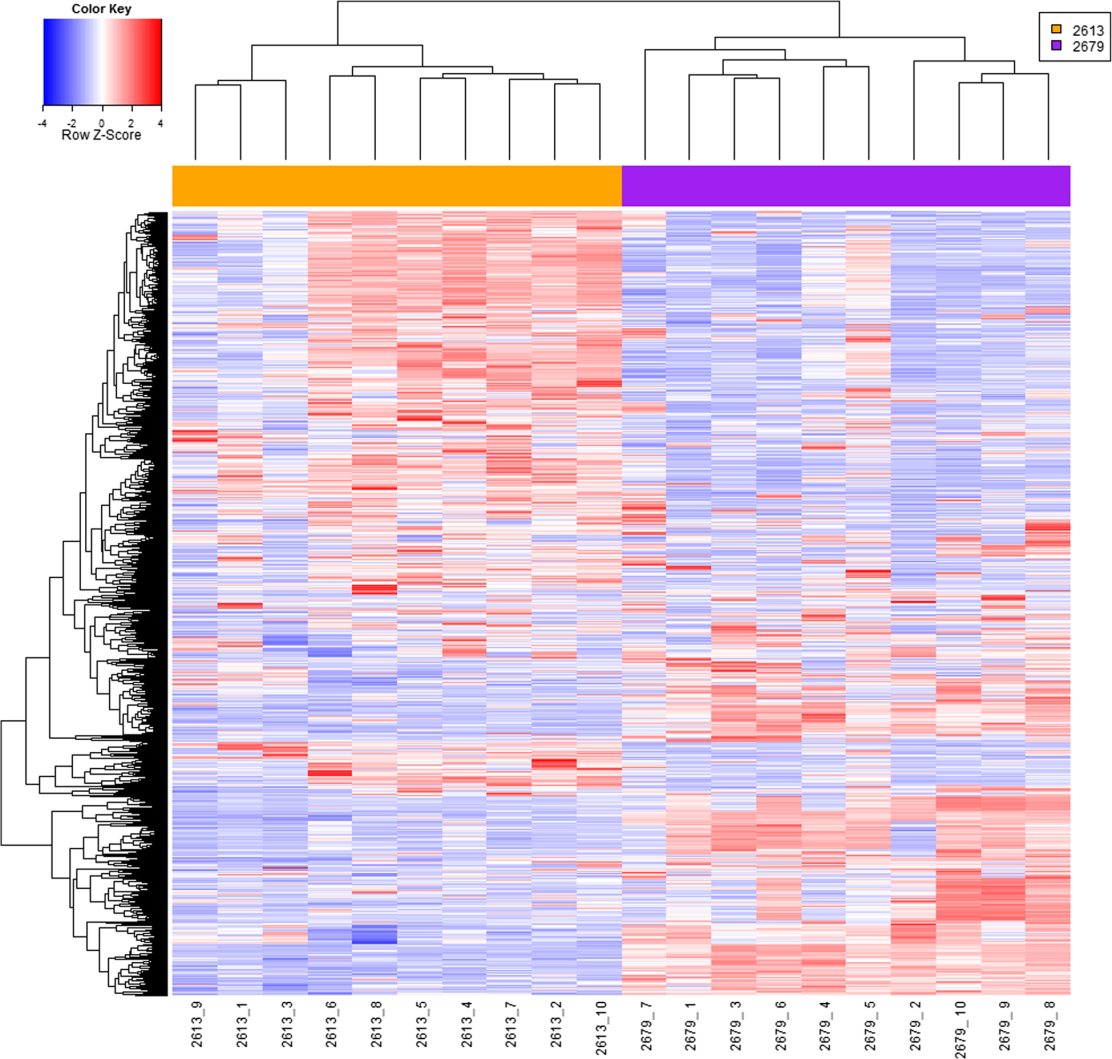
Cluster analysis of the top 1,000 most variable genes from each of 10 individual DTC isolated from the bone marrow of two PCa patients (2613 and 2679) with advanced metastatic disease.

**Figure 6:**
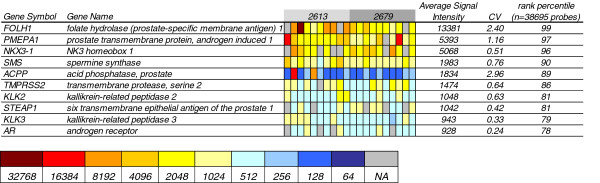
Heatmap of the top 10 expressed prostate specific genes from each of 10 individual DTC isolated from the bone marrow of two PCa patients (2613 and 2679) with advanced metastatic disease.

To validate the method we wished to determine if the methodology could be used in the analysis of individual tumor cells isolated from the bone marrow of patients. Similar to the C4-2B cell samples, 10 individual DTC from 2 patients were amplified. To determine if there was a difference in the input material from the patients relative to the cultured cells we assessed the amplified fragment sizes. The mean fragment size on the Bioanalyzer for 1 C4-2B cell was 610 (range 275–966), 5-cell 493 (range 144–968) and 10-cell 598 (range 178–1168). For the patient cells it was 193 (range 139–319) and 224 (range 127–469) respectively (we did not observe any associations between amplicon size and the gene expression array results). After amplification the samples were Alexa Fluor® 3 labeled single-cell samples were hybridized to Agilent 4x44k human genome microarrays along with Alexa Fluor® 5 labeled reference RNA. A total of 16,039 probes were positive on the patient sample arrays. Cluster analysis revealed similarities between the cells isolated within a patient, but not between patients (Figure 5). We then analyzed the expression of prostate specific genes from each of 10 individual DTC isolated from the two PCa patients (2613 and 2679). The heatmap in Figure 6 shows that each of the top 10 expressed prostate specific genes was in the top 75^th^ percentile of genes expressed in these samples. Additionally, to identify differences between biological pathways present in the two patient sample sets, using 10,731 known genes to compare the profiles of each patient by gene set enrichment analysis two gene sets were significantly upregulated with a false discovery rate <25% [15,16]. The two pathways were the TRKA pathway (binds nerve growth factor and is implicated in PCa proliferation) and the activation of the RAC pathway (associated with cell motility).

## Discussion

Herein we describe a one-step amplification of mRNA from a single cancer cell and hybridization to an oligonucleotide gene expression array using commercially available gene expression array technology. The amplification technology employed used a linear isothermal method to amplify mRNA. Unlike exponential amplification, this linear amplification approach is carried out by replication of only the original transcripts not replication of copies. An additional advantage is that the amplified cDNA can be used directly in RT-qPCR analysis. The commercially available kit used was employed based on availability at the start of these studies and does not compare this to other now commercially available kits using a similar platform. Using this microarray platform, approximately 76% of the probes detected in 10-cell pooled samples were detected in the single-cell samples. This decrease in probes detected and signal intensity appears to be related to the amount of material available. For those probes that were detected, we found a high correlation between the relative signal intensity of detected probes on arrays from single-cell and 10-cell pools. Additionally, we found a significant correlation between the single-, 5-, and 10-cell samples using gene set enrichment analysis.

Many groups have used PCR-based technologies to analyze a subset of genes from a single cell; however, screening of thousands of genes is more challenging. Other groups, notably Kurimoto et al. [10], have isolated a transcriptomic profile of individual cells; these methods are extremely effective but are not always available throughout the greater scientific community and are labor and time intensive. Similar methods to profile individual cells are also being developed; Islam et al. [17] have used highly multiplex RNA-seq to obtain a single-cell transcriptomic profile on a large number of single cells. Reproducibility was good; however, as in our study, the detection level decreased for genes expressed at lower levels. Additionally, they found a correlation of 0.86 for embryonic stem cells and 0.63 for embryonic fibroblasts compared to 0.86 for 10 pg of human brain RNA as a reference. Cann et al., have also used mRNA-Seq to profile gene expression in CTC [18]. Using Magsweeper technology to isolate LNCaP cells they found a high correlation in gene expression (R^2^ = 0.985) between Magsweeper and control pools of LNCaP cells. For LNCaP controls they measured 4622 ± 136 RefSeq transcripts with ≥10 FPKM. Of 67 CTC from 13 patients, 20 CTC from 4 patients had 2362 ± 865 RefSeq transcripts with ≥10 FPKM. Using an unsupervised cluster analysis the authors state that only two of 20 CTC did not cluster with other CTC from the same patient suggesting limited heterogeneity. However this may be limited by the number of samples tested and the number of transcripts available for analysis. While a smaller number of transcripts were available for analysis using this mRNA-Seq approach compared to the gene expression arrays reported here, there are other obvious benefits to using mRNA-Seq relative to gene expression analysis alone.

The importance of using a commercially available technology accessible to a broad user group is important as it provides the ability to examine individual cells to most laboratory based research groups. This is of particular interest to groups working on CTC/DTC, where the technology needs to go beyond measuring cell number to characterizing the molecular profile of individual cells [7,19]. This is particularly important as tumor cell heterogeneity could significantly impact the transcriptomic profile displayed by these cells at different times throughout the disease process [20]. Tumor cell heterogeneity may arise due to clonal evolution within a single tumor or multiple metastatic tumor sites shedding cells. This heterogeneity restricts our ability to obtain a definitive transcriptomic profile from CTC/DTC to further characterize the biological events occurring in response to treatment or the identification of tumor biomarkers. We have shown that a commercially available technology could be used to profile tumor cells grown in culture and cells isolated from the bone marrow of patients with PCa. These data show that the technology is available to obtain a significant and relevant gene expression profile from individual cells isolated from clinical biospecimens.

The caveats associated with the isolation of single cells from patient samples include: warm ischemia time, cell viability, and the RNase activity in cancer cells *in vivo* relative to somatic cells. While these parameters could all impact the quality of samples and therefore limit the transcriptomic profile observed, we have shown that a transcriptomic profile can be obtained from patient specimens. Additionally, examining tumor cell heterogeneity requires a greater number of single-cell samples to be analyzed from each patient; e.g., when profiling 10 cells from one patient, sampling 10 patients would now require 100 samples for analysis. This highlights the importance of a validated and efficient commercially available detection methodology that limits sample-to-sample variability and sample quality. A future challenge to increase the utility of this approach will be to automate the process of isolating cells of interest, possibly using microfluidic devices [21], and to provide a stage for direct RNA amplification or RNA-seq methodologies.

## Conclusions

While the amount of material available for amplification is restrictive limiting the number of positive probes on the single-cell arrays, we found good correlation between relative signal intensities of probes detected on single-cell arrays when compared to both 5- and 10-cell arrays. Therefore, obtaining a transcriptomic profile from a single cell in culture and from patient biospecimens using commercially available technology is feasible and potentially useful. In its current form this methodology can be used for research purposes; however, due to the dramatic potential increase in sample number per patient, the successful combination of this technology with novel high-throughput procedures will be required for clinical utility.

## Authors’ contributions

CM, RLV, CW, IC, RC, and BL conceived and designed the experiments. IC, RC, JX, RG, SC, CW, BL and CM analyzed the data. CW, IC, RC, BL, JX, SC, RLV, and CM wrote the manuscript. CW, RC, BL, SRL and CG performed the experiments. PHL, BM, PN provided materials and input into the project’s direction. All authors read and approved the final manuscript.

## Supplementary Material

Additional file 1: Table S1 Primer sequences, amplicon length, possible splice variants, 3’ bias, and primer specificity of the 10 genes examined by RT-qPCR in this study. Click here for file

Additional file 2: Table S2 Quantitative RT-PCR of 4 housekeeping genes (ACTB, GAPDH, YWHAZ, and GAPDH) for each of the 10 single-, 5-, and 10-cell samples. * Indicates removed from analysis based on quality control. NTC = No template control; na = no amplification detected after 46 cycles; 10 pg and 100 pg represents C4-2B total RNA from the same original culture. Click here for file

Additional file 3: Table S3 M ratio pair-wise correlation analysis for one cell. Based on the Dixon test for the correlation coefficients, the p-value for testing outliers is 0.457. Using p-value = 0.05 as cut-off. Array data: The microarray data for these experiments have been deposited in the Gene Expression Omnibus database (available at http://www.ncbi.nlm.nih.gov/geo) under accession number GSE38416. Click here for file

Additional file 4: Table S4 Gene Set Enrichment Analysis (GSEA) of single-, 5-, and 10-cell samples. GO = Gene Ontology; NES = Normalized Enrichment Score. The enrichment score reflects the degree to which the gene set is overrepresented at the extremes of the entire ranked list (n = 925). Click here for file
