# Supplementary material for: Single cell transcriptomic analysis of prostate cancer cells
Source: BMC Mol Biol. 2013 Feb 16;14:6. doi: 10.1186/1471-2199-14-6 (PMC3599075; doi:10.1186/1471-2199-14-6)
Supplement: Additional file 1: Table S1 — Primer sequences, amplicon length, possible splice variants, 3’ bias, and primer specificity of the 10 genes examined by RT-qPCR in this study. [file 1471-2199-14-6-S1.pdf]

Additional file 1: Table S1

NAME	SEQUENCE (5'-3')	PRODUCT LENGTH	POSSIBLE SPLICE VARIANTS	EXON-EXON BOUNDARY	EFFICIENCY
**ACPP-F**	CCCTCCTGGTTGGTCACAGAAT	101	no	no	103.0
**ACPP-R**	GAAAATGGACCCAAGGACAAACA				
**ACTB-F**	AGCCTCGCCTTTGCCGATCCG	100	no	yes	99.8
**ACTB-R**	ACATGCCGGAGCCGTTGTCGA				
**AR-F**	ATCCTCATATGGCCCAGTGTCAAG	102	variants	no	99.9
**AR-R**	GCTCTCTAAACTTCCCGTGGCATA				
**EPCAM-F**	ATCCAGAACTTGGACTCCATCG	113	no	no	97.2
**EPCAM-R**	GGCAGCTTTCAATCACAAATCA				
**FKBP5-F**	AAAAGGCCACCTAGCTTTTTGC	102	variants	no	102.0
**FKBP5-R**	CCCCCTGGTGAACCATAATACA				
**GAPDH-F**	TCTTTTGCGTCGCCAGCCGA	92	no	yes	98.7
**GAPDH-R**	ACCAGGCGCCCAATACGACC				
**KLK3-F**	GCATGGGATGGGGATGAAGTAAG	105	variants	no	101.3
**KLK3-R**	CATCAAATCTGAGGGTTGTCTGGA				
**RPL13A-F**	CCTGGAGGAGAAGAGGAAAGAGA	126	no	yes	98.4
**RPL13A-R**	TTGAGGACCTCTGTGTATTTGTCAA				
**TMPRSS2-F**	AGCAGGCTGGTTTGCAAGAA	96	variants	no	102.6
**TMPRSS2-R**	CAGACGGATCCTGCAAATGG				
**YWHAZ-F**	AGCTCCTCAAGAGCAGGGACAATGT	101	variants	no	95.2
**YWHAZ-R**	TCAAGACTCACTGCCTCCCATCATCA				
