# Supplementary material for: Single cell transcriptomic analysis of prostate cancer cells
Source: BMC Mol Biol. 2013 Feb 16;14:6. doi: 10.1186/1471-2199-14-6 (PMC3599075; doi:10.1186/1471-2199-14-6)
Supplement: Additional file 2: Table S2 — Quantitative RT-PCR of 4 housekeeping genes (ACTB, GAPDH, YWHAZ, and GAPDH) for each of the 10 single-, 5-, and 10-cell samples. * Indicates removed from analysis based on quality control. NTC = No template control; na = no amplification detected after 46 cycles; 10 pg and 100 pg represents C4-2B total RNA from the same original culture. [file 1471-2199-14-6-S2.pdf]

Additional file 2: Table S2

Sample ID	ACTB	GAPDH	YWHAZ	RPL13A	
NTC	39.60	42.10	na	na	
10 pg RNA	26.99	19.75	23.25	17.98	
100 pg RNA	20.25	19.54	19.61	15.78	
1 cell	26.44	30.70	27.67	22.21	
1 cell	27.71	29.65	27.19	20.68	
1 cell	44.60	42.90	na	33.00	*Excluded
1 cell	43.60	29.26	22.30	21.93	
1 cell	28.43	25.68	38.70	17.64	
1 cell	26.51	24.54	21.15	18.02	
1 cell	35.80	25.68	21.04	20.31	
1 cell	36.70	28.38	25.36	20.07	
1 cell	29.34	21.12	21.06	20.64	
1 cell	28.53	24.27	20.42	16.70	
Average	32.77	28.22	24.99	21.12	
5 cells	22.10	26.92	19.71	17.22	
5 cells	41.30	27.42	21.97	19.70	
5 cells	28.13	22.99	23.47	18.18	
5 cells	41.50	23.36	21.48	20.50	
5 cells	na	27.25	20.90	20.06	
5 cells	21.68	18.20	17.65	16.49	
5 cells	27.62	23.91	19.04	16.08	
5 cells	26.90	22.10	19.93	17.53	
5 cells	27.36	26.43	21.57	18.09	
5 cells	28.76	25.66	19.95	19.00	
Average	29.48	24.42	20.57	18.28	
10 cells	22.21	24.93	18.53	17.12	
10 cells	44.70	32.10	24.30	20.60	*Excluded
10 cells	23.58	19.88	20.08	16.70	
10 cells	26.85	22.66	19.87	17.86	
10 cells	25.25	22.34	21.05	17.76	
10 cells	24.72	20.99	19.23	16.08	
10 cells	25.13	20.89	17.44	14.64	
10 cells	22.60	21.19	18.74	16.43	
10 cells	23.89	23.63	18.64	16.03	
10 cells	25.95	22.72	18.38	16.85	
Average	29.43	25.70	21.81	18.90	
