# Supplementary material for: Single cell transcriptomic analysis of prostate cancer cells
Source: BMC Mol Biol. 2013 Feb 16;14:6. doi: 10.1186/1471-2199-14-6 (PMC3599075; doi:10.1186/1471-2199-14-6)
Supplement: Additional file 3: Table S3 — M ratio pair-wise correlation analysis for one cell. Based on the Dixon test for the correlation coefficients, the p-value for testing outliers is 0.457. Using p-value = 0.05 as cut-off. Array data: The microarray data for these experiments have been deposited in the Gene Expression Omnibus database (available at http://www.ncbi.nlm.nih.gov/geo) under accession number GSE38416. [file 1471-2199-14-6-S3.pdf]

Additional file 3: Table S3

Single Cell	#1	#2	#3	#4	#5	#6	#7	#8	#9	#10
#1	1.000	0.603	0.602	0.637	0.627	0.588	0.555	0.505	0.526	0.561
#2	0.603	1.000	0.585	0.623	0.603	0.640	0.568	0.516	0.490	0.573
#3	0.602	0.585	1.000	0.611	0.648	0.568	0.561	0.471	0.448	0.543
#4	0.637	0.623	0.611	1.000	0.623	0.605	0.571	0.532	0.522	0.581
#5	0.627	0.603	0.648	0.623	1.000	0.598	0.557	0.527	0.520	0.589
#6	0.588	0.640	0.568	0.605	0.598	1.000	0.563	0.512	0.491	0.571
#7	0.555	0.568	0.561	0.571	0.557	0.563	1.000	0.564	0.557	0.596
#8	0.505	0.516	0.471	0.532	0.527	0.512	0.564	1.000	0.548	0.552
#9	0.526	0.490	0.448	0.522	0.520	0.491	0.557	0.548	1.000	0.576
#10	0.561	0.573	0.543	0.581	0.589	0.571	0.596	0.552	0.576	1.000
