# Supplementary material for: Single cell transcriptomic analysis of prostate cancer cells
Source: BMC Mol Biol. 2013 Feb 16;14:6. doi: 10.1186/1471-2199-14-6 (PMC3599075; doi:10.1186/1471-2199-14-6)
Supplement: Additional file 4: Table S4 — Gene Set Enrichment Analysis (GSEA) of single-, 5-, and 10-cell samples. GO = Gene Ontology; NES = Normalized Enrichment Score. The enrichment score reflects the degree to which the gene set is overrepresented at the extremes of the entire ranked list (n = 925). [file 1471-2199-14-6-S4.pdf]

Additional file 4: Table S4

GO Gene Set Name	Gene Set Size	1 cell NES	5 cell NES	10cell NES
STRUCTURAL_CONSTITUENT_OF_RIBOSOME	80	3.82	4.05	3.91
RNA_BINDING	237	3.24	3.51	3.45
RIBONUCLEOPROTEIN_COMPLEX	129	2.96	3.20	3.18
MITOCHONDRION	334	3.03	3.23	3.16
MITOCHONDRIAL_PART	140	2.93	3.18	3.05
RNA_PROCESSING	154	2.90	3.12	2.98
TRANSLATION	176	2.87	2.90	2.94
RNA_SPLICING	82	2.87	3.01	2.91
MRNA_PROCESSING_GO_0006397	68	2.60	2.79	2.90
MITOCHONDRIAL_INNER_MEMBRANE	66	2.83	2.88	2.90
MITOCHONDRIAL_MEMBRANE	85	2.95	2.97	2.89
ORGANELLE_ENVELOPE	164	2.75	2.93	2.88
MITOCHONDRIAL_MEMBRANE_PART	52	2.97	2.95	2.88
RIBONUCLEOPROTEIN_COMPLEX_BIOGENESIS_AND_ASSEMBLY	79	2.90	3.03	2.87
ORGANELLE_INNER_MEMBRANE	74	2.88	2.89	2.86
ENVELOPE	164	2.79	2.91	2.86
MITOCHONDRIAL_ENVELOPE	95	2.85	2.96	2.85
MRNA_METABOLIC_PROCESS	78	2.69	2.84	2.85
STRUCTURAL_MOLECULE_ACTIVITY	235	2.78	2.97	2.84
ORGANELLE_MEMBRANE	290	2.76	2.92	2.83
ORGANELLE_LUMEN	436	2.74	2.81	2.80
MEMBRANE_ENCLOSED_LUMEN	436	2.75	2.82	2.78
RIBOSOME	39	2.58	2.86	2.76
PROTEIN_RNA_COMPLEX_ASSEMBLY	64	2.78	3.02	2.76
SPLICEOSOME	44	2.52	2.60	2.72
